# Determination of Sinomenine in Cubosome Nanoparticles by HPLC Technique

**DOI:** 10.1155/2015/931687

**Published:** 2015-02-05

**Authors:** Yanfang Zhou, Chunlian Guo, Hongying Chen, Yudai Zhang, Xinsheng Peng, Ping Zhu

**Affiliations:** ^1^Guangdong Medical College, Xincheng Avenue, Guangdong 523808, China; ^2^Department of Cardiovascular Surgery, Guangdong Cardiovascular Institute, Guangdong General Hospital, Guangdong Academy of Medical Sciences, Guangzhou 510100, China

## Abstract

We applied HPLC technique to quantitatively analyze sinomenine in cubosome nanoparticles. The chromatographic method was performed by using an isocratic system. The mobile phase was composed of methanol-PBS(pH6.8)-triethylamine (50 : 50 : 0.1%) with a flow rate of 1 mL/min; the detection wavelength was at 265 nm. Sinomenine can be successfully separated with good linearity (the regression equation is *A* = 10835*C* + 1058; *R* ^2^ = 1.0) and perfect recovery (102.2%). The chromatograph technique was proper for quality control of sinomenine in cubosome nanoparticles.

## 1. Introduction

**Figure 1:**
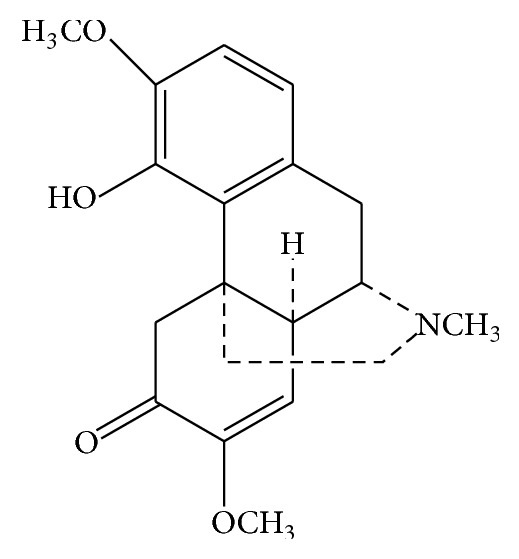
The structure of sinomenine.

Sinomenine (7,8-didehydro-4-hydroxyl-3,7-dimethoxy-17-methylmorphinan-6-one, chemical structure is shown in Figure 1) is an alkaloid isolated from the stem and root of Chinese medical plant* Sinomenium acutum* Rehd. et Wils. Because of its analgesic and anti-inflammatory effects, sinomenine has been utilized clinically to treat rheumatoid arthritis and neuralgia [1, 2].

In order to prevent the negative gastrointestinal adverse reactions and low bioavailability of sinomenine oral preparations, researchers have been trying to use transdermal drug delivery system to overcome these shortcomings. For example, microemulsion based gel [3], liposome [4], and ethosomes [5] had been documented in the literature. We concerned the development of sinomenine preparations for a long time and found cubosome, a novel kind of transdermal drug delivery system, could be very well suitable to the sinomenine. Cubosome and liposome both had the bilipids membrane, but cubosome showed better storing stability compared to liposomes [6]. Cubosome could also enhance the drug deposition in the skin and showed excellent skin-targeted characteristics [7]. Our previous experiment has proved cubosome could enhance sinomenine skin permeation (7-fold compared to gel) and a patent was applied in China (ZL 201310087778.6). Here we describe the determination of sinomenine in cubosome nanoparticles by HPLC technique.

## 2. Experiments

### 2.1. Reagents and Chemicals

Glycerol monooleate (DIMODAN MO/D KOSHER, material number 116703) was kindly provided by Danisco Cultor (Brabrand, Denmark). Poloxamer 407 (PEO98POP67PEO98) was a gift from BASF (Ludwigshafen, Germany). Sinomenine was purchased from Shenzhen Yihao Technology Development Co., Ltd. (Guangdong, China). Methanol (Chromatographic Grade) was purchased from Tianjin Fuyu Chemical Co., Ltd. (Tianjin, China). Water purified through a flow water purification system (Qingdao, China) was used throughout this study. PBS (pH 6.8) was made according to the Chinese Pharmacopoeia (2010). All other reagents were of analytical grade.

### 2.2. Apparatus

Analyses were performed on UV-6000s Spectrophotometer (Shanghai Metash Instruments Co., Ltd.) and LC-20AT High Performance Liquid Chromatograph with SPD-20A UV-detector (Shimadzu (Suzhou) Instruments Co., Ltd).

### 2.3. Preparation of Sinomenine Cubosome [7, 8]

Firstly, glycerol monooleate (2.7 g) and poloxamer 407 (0.3 g) were first melted at 60°C in a hot water bath until they were homogeneous, after which sinomenine (0.3 g) was added to blend under continuous stirring. Water (6.7 mL) was then added gradually and the mixture was vortex-mixed to achieve a homogeneous state. After equilibration for at least 48 hours at room temperature, the cubic phase gel was formed. By adding 20 mL of water, the cubic gel was disrupted by mechanical stirring. Subsequently, the crude dispersion was fragmented for 10 min by intermittent probe sonication. The final dispersion of cubic nanoparticles was stored at room temperature for later studies.

### 2.4. Chromatographic Conditions

The analytical column was an Hichrom C18 column (4.6 × 250 mm, 5 *μ*m, inner diameter, 5 *μ*m). The mobile phase was methanol-PBS(pH6.8)-triethylamine (50 : 50 : 0.1%) with a flow rate of  1 mL/min, and the detection wavelength was at 265 nm. The sample injection volume was 10 *μ*L. The column temperature was maintained at 25°C.

### 2.5. Preparation of Standard Solutions

Sinomenine stock solution was prepared in 50% methanol solution and sonicated for 5 min to obtain stock solution concentrations of 320 *μ*g/mL. This solution was further diluted with 50% methanol solution to yield solutions containing 160.0, 80.0, 40.0, 20.0, 10.0, 5.0, 2.5, and 1.25 *μ*g/mL.

### 2.6. Sample Preparation Procedure

0.2 mL (approximately 200 mg) of cubosome nanoparticles was accurately transferred into a 10 mL volumetric flask, dissolved, and made up to volume with methanol. Then, the sample solutions were filtered using a 0.45 *μ*m filter membrane and 10 *μ*L aliquot was injected into the HPLC system.

## 3. Results and Discussion

### 3.1. Preparation of Sinomenine Cubosome

**Figure 2:**
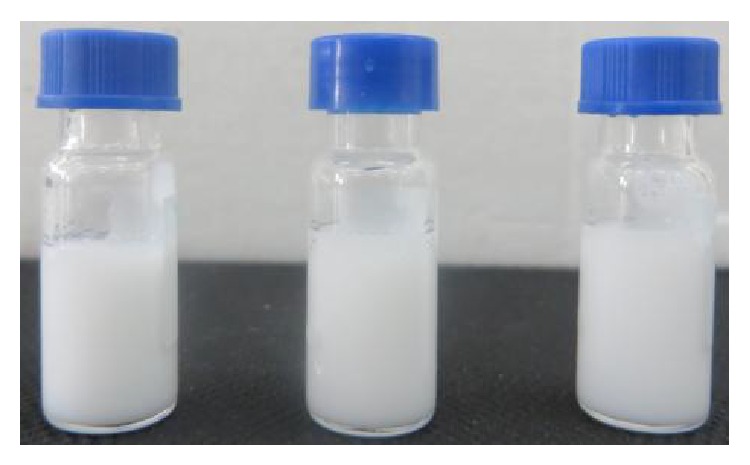
Photograph of sinomenine cubosome.

**Figure 3:**
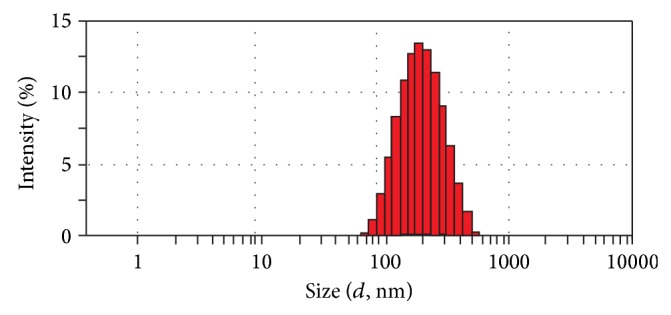
Size distribution of sinomenine cubosome.

The milky coarse dispersions of phytantriol-based sinomenine cubosomes were obtained (Figure 2). The cubosomal particle size was determined by photon correlation spectroscopy using a ZetasizerNano ZS90 (Malvern Instruments Malvern, UK) at 25°C. The mean *z*-average diameter and polydispersity indices (PDI) were obtained by cumulative analysis using the MALVERN software. The mean diameter of different cubosome dispersions was in an approximate range of 177.8 nm with the polydispersity indices (PDI) value of 0.158 (Figure 3).

### 3.2. Chromatographic Conditions

**Figure 4:**
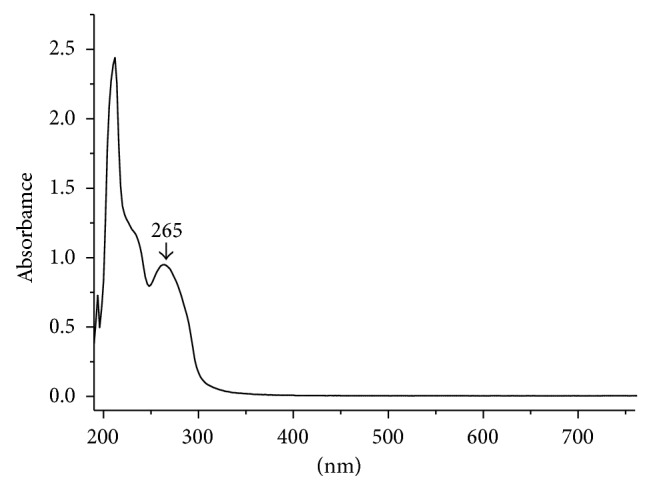
UV spectrum of sinomenine.

UV spectrum of sinomenine showed maximum absorbance at 265 nm wavelength (Figure 4). Therefore, 265 nm was selected as the detection wavelength. In selected spectrometry conditions, sinomenine chromatography's symmetry was with higher degrees. The response was high and the retention time was less than 10 min with good reproducibility.

### 3.3. Specificity

**Figure 5:**
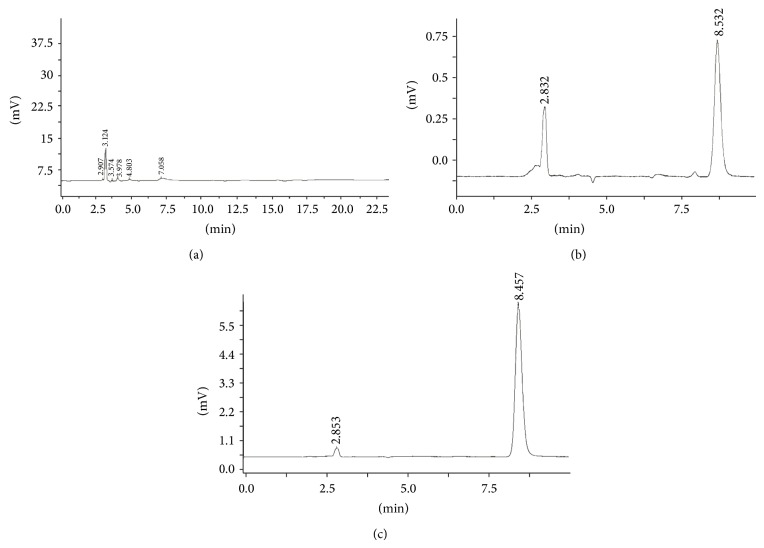
HPLC chromatogram of blank cubosome (a), standard sinomenine (b), and sinomenine cubosome (c).

Blank cubosome sample was prepared according to Section 2.3 (no sinomenine), and the chromatogram was shown in Figure 5(a). A certain concentration of sinomenine standard solution and sinomenine cubosome sample solution were injected into the HPLC system with the same operation, and the chromatogram was shown in Figures 5(b) and 5(c). The retention time was 8.53 min. Obviously, the peaks of the sinomenine were well separated and were not affected by the excipients.

### 3.4. Linearity

The calibration curves for sinomenine were found to be linear within the range of 1.25 to 320.0 *μ*g/mL. The regression equation was *A* = 10835*C* + 1058  (*R* ^2^ = 1.0), where *A* is peak area and *C* is the concentration (*μ*g/mL) of sinomenine standard solution. The correlation coefficient indicated a good linear relationship between peak area and concentration over a wide range.

### 3.5. Precision

Precision was demonstrated at 3 concentration levels in intraday and interday studies. Intraday precision was determined by injection of sinomenine standard solutions on the same day. Interday precision was checked by repeating the studies on two different days. The intraday and the interday precisions of sinomenine are summarized in Table 1. The RSD is found to be acceptable (RSD < 2%).

### 3.6. Recovery

Mean recovery for sinomenine at different concentration levels was found to be 100.3 ± 2.2% (RSD = 2.19%, *n* = 3), 103.1 ± 2.2% (RSD = 2.2%, *n* = 3), and 103.3 ± 1.7% (RSD = 1.6%, *n* = 3), respectively. The low values of RSD revealed the present method was accurate, reliable, and reproducible.

### 3.7. Limits of Detection (LOD) and Quantification (LOQ)

The LOD (signal/noise ratio of 3 : 1) and LOQ (signal/noise ratio of  10 : 1) were determined as 7.8 × 10^−2^ *μ*g/mL and 2.6 × 10^−1^ *μ*g/mL, respectively.

### 3.8. Content of Sinomenine in Cubosome Nanoparticles

Sinomenine was determined with the proposed method in cubosome nanoparticles. The mean concentration was 8.0 mg/mL (RSD = 1.9%, *n* = 3).

### 3.9. The Stability Experiment

**Figure 6:**
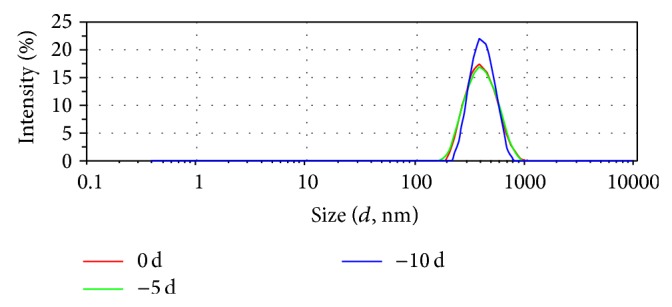
The influence on cubosome size distribution by high temperature test.

The stability experiment was aimed at testing the effects during storage. The stress testing showed the cubosome was physically stable under high temperature (60°C) within 10 days (Figure 6). It was similar to the literatures in which cubosomes showed better storing stability at room temperature and could endure heat treatment compared to liposome [9–11]. That also provided the base for the practicability and scientificalness of the formulations and process parameters.

## 4. Conclusions

In this work, we used HPLC method to separate and determine the sinomenine in cubosome nanoparticles. The linear range of sinomenine concentration was 1.25 to 320.0 *μ*g/mL. Calculated by samples, the intra- and interassay precision (RSD) were less than 2%. The average recovery rate was 102.3%. Sinomenine peaks were well resolved and free from tailing (<1.5). This method was sensitive with good precision and accuracy. The separation time was 8.53 min and the excipients did not interfere with the detection of sinomenine. This method was applied to sinomenine cubosome researches.

## Figures and Tables

**Table 1:** The intraday and the interday precision (*n* = 3).

Compound	Conc. (*μ*g/mL)	Intraday		Interday	
		Mean conc. (*μ*g/mL)	RSD (%)	Mean conc. (*μ*g/mL)	RSD (%)
Sinomenine	100	101.3	1.33%	101.9	1.51%
	150	149.6	1.026%	151.8	1.84%
	200	200.9	1.014%	199.6	1.36%
